# Retrotransposon Domestication and Control in *Dictyostelium discoideum*

**DOI:** 10.3389/fmicb.2017.01869

**Published:** 2017-10-05

**Authors:** Marek Malicki, Maro Iliopoulou, Christian Hammann

**Affiliations:** Ribogenetics Biochemistry Lab, Department of Life Sciences and Chemistry, Jacobs University Bremen, Bremen, Germany

**Keywords:** Skipper-1, DIRS-1, TRE5-A, RNA interference, retrovirus

## Abstract

Transposable elements, identified in all eukaryotes, are mobile genetic units that can change their genomic position. Transposons usually employ an excision and reintegration mechanism, by which they change position, but not copy number. In contrast, retrotransposons amplify via RNA intermediates, increasing their genomic copy number. Hence, they represent a particular threat to the structural and informational integrity of the invaded genome. The social amoeba *Dictyostelium discoideum*, model organism of the evolutionary Amoebozoa supergroup, features a haploid, gene-dense genome that offers limited space for damage-free transposition. Several of its contemporary retrotransposons display intrinsic integration preferences, for example by inserting next to transfer RNA genes or other retroelements. Likely, any retrotransposons that invaded the genome of the amoeba in a non-directed manner were lost during evolution, as this would result in decreased fitness of the organism. Thus, the positional preference of the *Dictyostelium* retroelements might represent a domestication of the selfish elements. Likewise, the reduced danger of such domesticated transposable elements led to their accumulation, and they represent about 10% of the current genome of *D. discoideum*. To prevent the uncontrolled spreading of retrotransposons, the amoeba employs control mechanisms including RNA interference and heterochromatization. Here, we review TRE5-A, DIRS-1 and Skipper-1, as representatives of the three retrotransposon classes in *D. discoideum*, which make up 5.7% of the *Dictyostelium* genome. We compile open questions with respect to their mobility and cellular regulation, and suggest strategies, how these questions might be addressed experimentally.

## Transposable elements and their habitat

A significant fraction of all eukaryotic genomes is scattered with repetitive sequences that are predominantly related to different types of transposable elements (TEs) (Biscotti et al., 2015). At first glance, TEs are selfish or parasitic DNA, imposing the burden of their propagation on the invaded host. However, their ability to move and multiply within host genomes can not only have a negative impact, but can also improve the fitness of their host (McClintock, 1950). It is well established that transposition can alter gene expression, as TE insertion upstream of a gene can lead to its up-regulation, and downstream insertion to its down-regulation (Slotkin and Martienssen, 2007). Furthermore, transposition can promote inversions and deletions of large chromosomal DNA fragments, gene mutation, gene shuffling, transcriptional regulation, dispersion of regulatory sequences, genomic recombination and chromosomal rearrangements (Huang et al., 2012). Thus, TE mobility is a crucial factor driving genome evolution (Gbadegesin, 2012).

**Figure 1:**
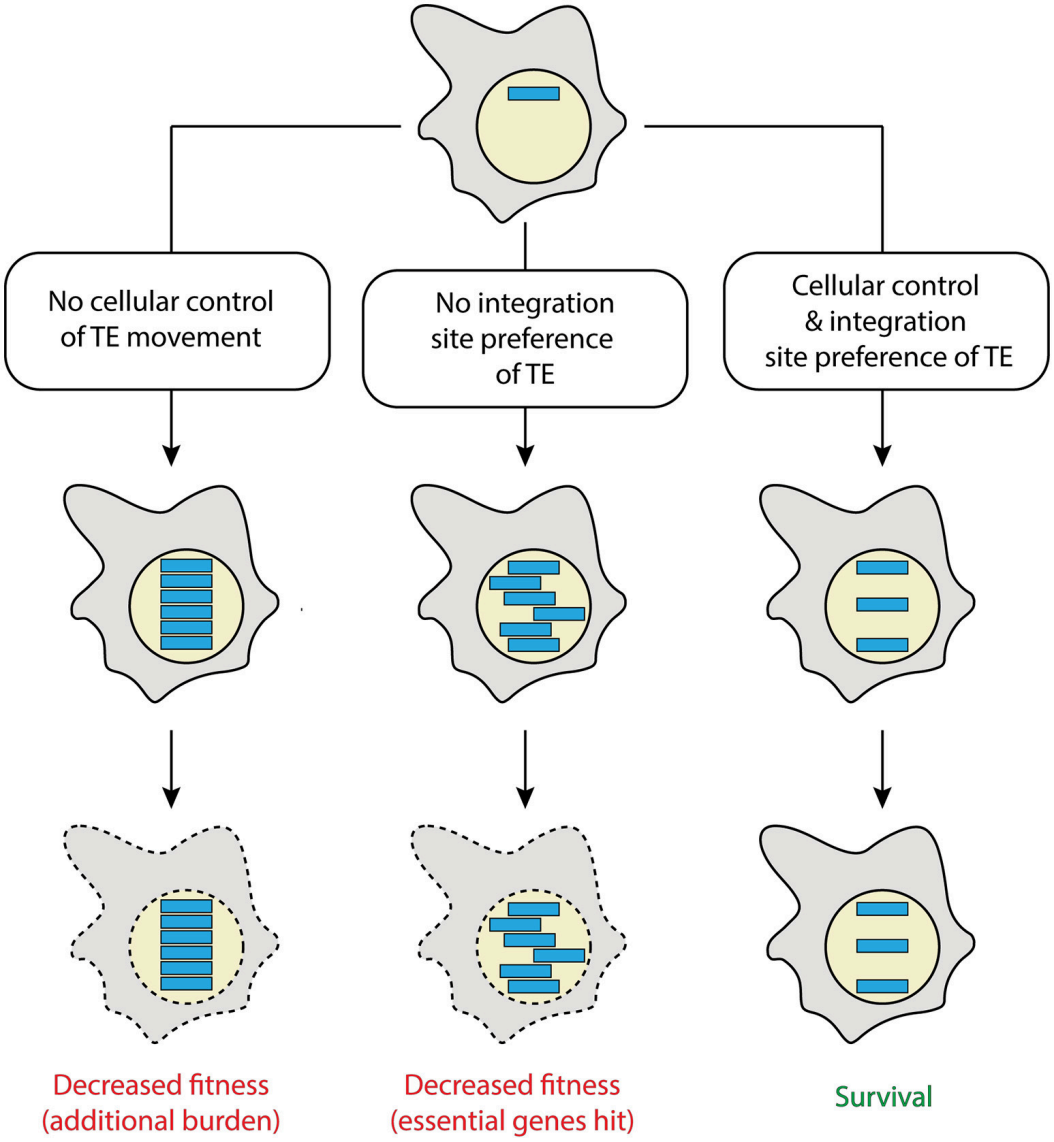
Possible fates of transposable elements. Shown is a cell (gray) with a nucleus (yellow), in which a transposable element (TE; blue) invaded. Depending on the TE features and its control by cellular elements, different scenarios can be envisaged. Lack of cellular control of the TE will lead to its significant accumulation, posing additional burden to the host (left). Random integration (center) by lack of TE site preference is prone to hit essential genes. Compared to a cellularly controlled TE that additionally was domesticated by displaying a safe integration site preference (right), the former two scenarios would result in cells with decreased fitness. This is expected to eventually lead to their loss during evolution (indicated by dashed lines). A combination of the first two scenarios (not shown) is thought to result in a particularly disfavored cell.

The natural urge of TEs to move is confronted with the pressure of their hosts to preserve the genomic integrity, with respect to both, structural and informational stability, a prerequisite to eventually replicate successfully (Figure 1). Therefore, as computational modeling suggests, the inhabitation of a genome by TEs happens in two stages: After invasion, for example by horizontal transfer of the TE, an initial transposition burst occurs. To reduce the burden of additional TE copies, the invaded host organism subsequently develops strategies to tightly control TE movement within the genome (Le Rouzic and Capy, 2005). Such regulatory mechanisms employ frequently parts of the RNA interference (RNAi) and epigenetic machineries (Castel and Martienssen, 2013). TE control is often incomplete such that TEs that integrate at random positions might cause deleterious mutations. This results in reduced fitness and might lead to the death of the organisms and thus disappearance of the TE (Figure 1). To counteract this threat, several selfish TEs have developed strategies to limit the potential deleterious consequences of their activity. One of them is to target gene-poor or transcriptionally inactive regions of the genome, like telomeres and centromeres (Levis et al., 1993; Gao et al., 2015), which essentially results in their domestication (Figure 1). Hence, similar to intracellular pathogens, TEs propagate within their host's genome while exploiting cellular mechanisms to maintain integrity and functionality of their niche.

## Two classes of transposable elements: retrotransposons and DNA transposons

In principle, mobile genetic elements are categorized on mechanistic grounds, distinguishing retrotransposons from DNA transposons (Kazazian, 2004; Goodier and Kazazian, 2008). The latter, called class II elements (Wicker et al., 2007), move usually by a cut-and-paste process mediated by a TE-encoded recombinase or transposase. They consist of inverted terminal repeats and at least one open reading frame (ORF) coding for a transposase, which excises the entire transposon allowing for integration into a new locus (Vos et al., 1996). Beyond the transposase gene, class II TEs are usually not transcribed, thus do not move through a full RNA intermediate (Wicker et al., 2007), and therefore are here not further considered.

In contrast to class II TEs, retrotransposons amplify through an RNA intermediate (Wicker et al., 2007). This RNA intermediate is converted to a complementary DNA (cDNA) molecule by a reverse transcriptase (RT) activity, followed by integration of resulting cDNA copies. Thus, these class I elements use a copy-and-paste mechanism that, in the absence of suitable control mechanisms, progressively increases their copy number in the genome (Castro-Diaz et al., 2015). Retroelements can be further divided into long terminal repeat (LTR) and non-LTR retrotransposons. The former have arisen from retroviruses that once integrated into the genome of the host or its ancestor. This can be inferred from their similar structural organization, with all or parts of the prototypic retrovirus coding sequences flanked by two LTRs (Friedli and Trono, 2015). Non-LTR elements are subdivided into autonomous and non-autonomous TEs. The autonomous elements have usually two ORFs that encode the proteins required for transposition. These can also act *in trans* on non-autonomous TEs, if these still have the sequences necessary for transposition (Wicker et al., 2007; Kapitonov and Jurka, 2008). In addition, autonomous retroelements are also categorized based on the enzyme that performs the integration: YR elements encode a tyrosine recombinase, while other retroelements employ integrases or endonucleases (Goodwin and Poulter, 2001; Duncan et al., 2002; Wicker et al., 2007).

## *Dictyostelium discoideum* and its mobile genetic elements

*Dictyostelium discoideum* serves as a model organism helping to understand biological phenomena like motility, chemotaxis, phagocytosis and cytokinesis (Unal and Steinert, 2006; Calvo-Garrido et al., 2010; Surcel et al., 2010; Cai and Devreotes, 2011). The amoeba belongs to the evolutionary supergroup of Amoebozoa (Adl et al., 2012), and it likely is its best-studied representative. In response to starvation, *Dictyostelium* forms differentiated multicellular structures upon aggregation of thousands of solitary amoebae (Fets et al., 2010). At the genomic level, *D. discoideum* amazed the research community with an unexpected diversity of mobile genetic elements (Glöckner et al., 2001) in a gene-dense arrangement (Eichinger et al., 2005). Roughly two thirds of its genome (34 Mb) are protein-coding genes, and 10% are TEs (Glöckner et al., 2001; Eichinger et al., 2005). In such a gene-dense genome, a high frequency of TEs appears unlikely to persist during evolution, unless (a) the TEs have developed strategies for damage-free transposition, (b) the invaded host has developed measures to control TE mobility, or (c) the host benefits from the presence of the TEs. In view of the genome composition of *D. discoideum*, it appears likely, that both the host and the invading TEs have developed strategies to allow for co-existence, possibly even a mutually beneficial co-evolution.

The TEs in the *D. discoideum* genome have been charted in seminal work by Glöckner et al. (2001). Its DNA transposons represent 1.5% of the genome content and interestingly, none of their transposases share significant similarity with known transposases (Winckler et al., 2011). They fall into three main families, the Tdd elements, the DDT elements and the Thug elements with genomic frequencies of 0.5, 0.9, and 0.1%, respectively. To our knowledge, for none of these DNA transposons, expression or cellular control have been studied in detail. It would be of particular interest to analyze whether any of the control mechanisms that begin to emerge for the retrotransposons, discussed below, might also act on DNA transposons. The genome of *D. discoideum* contains retrotransposons that fall into three major classes: non-LTR, LTR, and YR retrotransposons (Glöckner et al., 2001; Winckler et al., 2011). Although the YR retrotransposons feature LTRs, they are considered their own class due to unique characteristics, like the presence of a tyrosine recombinase (Poulter and Goodwin, 2005). In *D. discoideum*, retrotransposons make up about 8% of the genome, which is a significant expansion compared to other dictyostelid genomes (Spaller et al., 2016). In recent years, representatives of each class of *D. discoideum* retrotransposons have been investigated, as detailed next.

## The non-LTR retrotransposons

**Figure 2:**
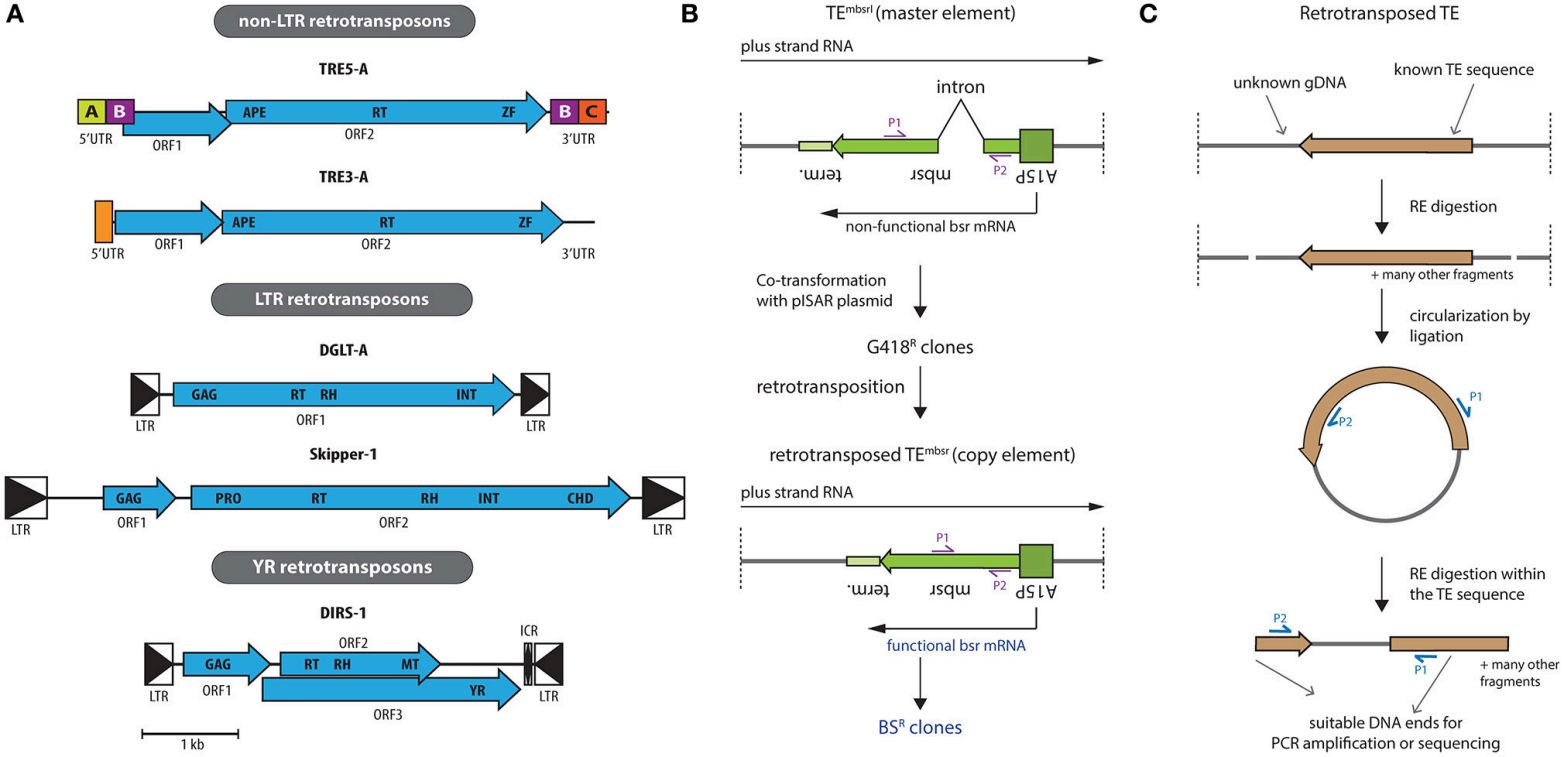
Retrotransposons encoded in the *D. discoideum* genome and strategies for their investigation. **(A)** Shown are the structures of the consensus elements with open reading frames (ORFs) in blue, drawn to scale (see bottom). The non-LTR retrotransposon family is separated into two subgroups, namely TRE5 and TRE3 (for 5′ and 3′ tRNA gene targeted retroelement, respectively), based on their integration preferences upstream or downstream of tRNA genes. Typical for all TRE elements is the presence of two ORFs, where the second one encodes for an apurinic/apyrimidinic endonuclease (APE), a reverse transcriptase (RT), and a zinc-finger domain (ZF). The bordering short untranslated regions (UTR) are indicated, which contain in the TRE5 subfamily distinct modules A–C. The LTR retrotransposons represent Ty3/gypsy-like elements that are surrounded by directed long terminal repeats (LTR, boxed triangles). DGLT-A has a single ORF encoding for a group-specific antigen (GAG) protein with a zinc finger-like signature followed by RT, ribonuclease H (RH), and integrase (INT) domains. Skipper-1 contains two ORFs. ORF1 codes for a GAG protein with a defined zinc finger-like motif. ORF2 codes for a protease (PRO), RT, RH, INT. Additionally, ORF2 of Skipper-1 contains a chromo domain (CHD) at the C-terminus. Note that all copies of DGLT-A and Skipper-2 in the genome of *D. discoideum* are incomplete, contrary to other dictyostelids (Spaller et al., 2016). DIRS-1 is the founding member of the class of tyrosine recombinase (YR) retrotransposons. Its three ORFs are surrounded by inverted LTRs. ORF1 encodes a putative GAG protein, ORF2 overlaps with ORF3 and encodes RT, RH and methyl transferase (MT) domains, while ORF3 contains the YR. **(B)** Schematic representation of the retrotransposition assay with a genetically tagged transposable element TE^mbsrI^ (mbsrI: minus-strand blasticidin S resistance (bsr) gene disrupted by an inverse intron), as applied successfully in the investigation of *Ty1* from *Saccharomyces cerevisiae*, mammalian LINEs and TRE5-A from *D. discoideum* (Boeke et al., 1985; Esnault et al., 2000; Ostertag et al., 2000; Siol et al., 2011). Shown is the orientation of the resistance cassette (referred to as “*master element*” when disrupted by an intron) with respect to the TE sequence, in which the cassette is embedded. A15P denotes the actin15 promoter and terminator sequence (term.) is shown. Upon co-transformation with a marker plasmid, e.g., pISAR, which confers G418 resistance to *D. discoideum*. Transformants with stably integrated plasmids are selected with G418. The *master element* is not able to generate blasticidin S (BS) resistant clones, because the respective gene is inactivated by an intron (from the *S17* gene, 74 bp in size). After a complete retrotransposition cycle, however, the intron is spliced out, resulting in TE^mbsr^ (minus-strand bsr gene), referred to as “*copy element*” upon loosing the intron in the resistance gene sequence. This enables expression of a functional bsr messenger RNA (mRNA), thereby conferring BS resistance (BS^R^). BS resistant clones thus are cells, in which at least one full retrotransposition cycle of the TE under investigation has been performed. Primers P1 and P2 can be used to confirm the splicing of the intron by PCR, and the size of the PCR product is indicative for the *master element* or the *copy element*. **(C)** Mapping of novel integration sites using tagged TEs. To address integration sites, genomic DNA (gDNA) is digested using restriction enzymes (RE) that cut outside the TE. Upon circularization of resulting fragments by ligation and RE digestion inside the TE sequence, linear fragments are obtained. These can be analyzed by PCR employing primers P1 and P2, or directly subjected to next generation sequencing.

The genome of the amoeba features a comparably large number of 418 transfer RNA (tRNA) genes. The two subfamilies of non-LTR retrotransposons in *Dictyostelium* target the up- and downstream regions of tRNA genes and have accordingly been named TRE5 and TRE3. The TREs represent 3.6% of the genome content (Glöckner et al., 2001). Both TRE subfamilies contain two ORFs (Figure 2A). A distinct integration distance of about 50 bp upstream and about 100 bp downstream of tRNA genes is observed for TRE5 and TRE3, respectively.

TRE5-A was the first identified TRE in the genome of *D. discoideum* (Marschalek et al., 1989; Winckler, 1998) and is understood best, mainly by work in the Winckler lab. The autonomous TRE5-A.1 contains two overlapping ORFs and three regulatory sequence modules A, B, and C (Figure 2A). TRE5-A ORF1 protein (ORF1p) physical interacts with subunits of the tRNA-gene specific transcription factors IIIB (TFIIIB), indicating that it might be involved in target site selection (Chung et al., 2007). Although it lacks sequence homology to ORF1p of other non-LTR retroelements (Glöckner et al., 2001), there is a certain functional correlation with ORF1p in the mammalian TE L1, which is also involved in genomic integration, though not at tRNA genes (Kolosha and Martin, 2003; Martin et al., 2008). Regulatory sequences of tRNA genes, in particular B-boxes at their 5′ end, are sufficient for TRE5-A targeting, even in the absence of a tRNA gene (Siol et al., 2006b). As TFIIIB binds to these sequences, it seems plausible that TRE5-A has hijacked RNA polymerase III transcription factors for its targeting.

ORF2 encodes for a polyprotein containing the enzymatic activities that are required for the retrotransposition cycle (Figure 2A; Winckler et al., 2011). The regulatory A module has RNA polymerase II promoter activity, the B module harbors the translation start site for ORF1 and the C module is required for retrotransposition (Marschalek et al., 1992; Schumann et al., 1994; Siol et al., 2011). The C module also harbors an internal promoter for the generation of antisense transcripts (Schumann et al., 1994). In recent years, the Winckler lab has established a genetically traceable version, TRE5-A^bsr^, schematically shown in Figure 2B. This proved most helpful in studying various aspects of TRE5-A retrotransposition (Siol et al., 2011). The A module could be replaced by an artificial promoter, indicating that this is its sole function. TRE5-A^bsr^ is based on the non-autonomous TRE5-A.2 variant, which lacks the ORF2 sequence, but is mobilized *in trans* by the ORF2p of endogenous TRE5-A.1 (Beck et al., 2002). Subsequently, cloning and sequencing of *de novo* integration sites of TRE5-A^bsr^ revealed the authentic positioning around 50 nucleotides upstream of tRNA genes (Beck et al., 2002; Siol et al., 2006a). Additionally, this construct was also instrumental to realize that, in principle, all tRNA genes can be targeted (Spaller et al., 2017). Whether integration alters tRNA gene expression cannot be easily investigated due to their high abundance and redundancy in *D. discoideum*. Unexpected TRE5-A^bsr^ integration sites in the extrachromosomal rDNA palindrome of the amoeba (Sucgang et al., 2003) were also uncovered, which are characterized by perfect B box sequences (Siol et al., 2011). Subsequently, additional integration sites in the vicinity of the RNA polymerase III-transcribed ribosomal 5S gene were characterized (Spaller et al., 2017). Taken together, these data strongly point towards a general coupling of TRE5-A integration with active RNA polymerase III transcription. As such, integration of TRE5-A would depend on the presence of regulatory A/B box sequences of tRNA genes, rather than the tRNA genes themselves.

Additionally, a host factor (CbfA for C-module binding Factor A; Geier et al., 1996) supports active TRE5-A retrotransposition, by stabilizing or upregulating TRE5-A sense and antisense transcripts (Bilzer et al., 2011). This transcription factor is also essential for the multicellular development of *D. discoideum* by transcriptionally activating the aggregation-specific adenylyl cyclase ACA (Winckler et al., 2004; Siol et al., 2006b). Later, CbfA was characterized as a general transcriptional regulator, with more than 1000 genes being differentially regulated at least 3-fold in a strain with largely reduced CbfA protein amounts. Amongst these was *agnC*, the gene encoding the Argonaute protein C, which experienced a more than 200-fold upregulation (Schmith et al., 2013). Argonaute proteins are key components of the RNAi machinery (Hutvagner and Simard, 2008), and therefore, this observation was of particular interest, as it opened the possibility that TRE5-A might be regulated by RNAi components. This holds particularly true, as complementary sense and antisense TRE5-A RNAs are present (Bilzer et al., 2011). Next to Argonaute proteins, RNA-dependent RNA polymerases (RdRPs) and Dicer proteins are key components of RNAi, and several representatives of these families exist in *D. discoideum* (Martens et al., 2002; Kuhlmann et al., 2005; Boesler et al., 2014; Wiegand et al., 2014; Kruse et al., 2016; Meier et al., 2016). Indeed, TRE5-A was found overexpressed in an *agnC* deletion strain, and downregulated in an AgnC overexpressing strain, resulting in a reduced retrotransposition rate (Schmith et al., 2015). No indication was found for an involvement of the three RdRPs of the amoeba, nor of its two Dicer proteins. This suggests that a distinct, AgnC-dependent RNAi pathway controls TRE5-A amplification, which, however, is counteracted by CbfA, resulting effectively in an active TRE5-A population in wildtype *Dictyostelium* (Beck et al., 2002; Siol et al., 2006a).

## The LTR retrotransposons

The *D. discoideum* genome features two related LTR retrotansposon families, Skipper and DGLT-A (Spaller et al., 2016). Both are Ty3/gypsy-like retotransposons that share the enzymatic activities required for retrotransposition (Figure 2A). These are, however, organized as one ORF in DGLT-A, and spread over two ORFs in Skipper. The *D. discoideum* genome does not feature any full-length DGLT-A copies, indicating that the TE might no longer be able to amplify (Winckler et al., 2005). Intriguingly, the DGLT-A elements in *D. discoideum* are also found 13–33 bp upstream of tRNA genes. Thus, two unrelated TEs, DGLT-A and TRE5 both integrate upstream of tRNA genes, indicating convergent evolution. A recent study expanded this view: in the evolution of dictyostelids, selection of tRNA genes as TE target was invented independently at least six times (Spaller et al., 2016).

Skipper is distinct from DGLT-A not only by the structural organization of its ORFs, but also by the presence of a chromo domain (CHD). Recent data indicated that Skipper retrotransposons in dictyostelids come in two varieties. Skipper-1 contains a conventional CHD and is found as largely fragmented elements in centromeric regions of the chromosomes (Glöckner and Heidel, 2009); also the related DGLT-P element harbors a CHD, resulting in a name change to Skipper-2, despite the fact that this CHD has somewhat diverged. Skipper-2 is found downstream of tRNA genes, similar to the TRE3 elements, another example of the convergent evolution of this target selection (Spaller et al., 2016).

CHDs are known to target retrotransposons to heterochromatin (Gao et al., 2008). In line with this, centromeric sequences in *D. discoideum* are characterized by heterochromatic H3K9 methylation marks (Kaller et al., 2007), and Skipper-1 co-localizes with the centromeric histone variant cenH3 (Dubin et al., 2010). At present, it is unknown though, whether centromeric Skipper-1 targeting is an active CHD-mediated process. Alternatively, the apparent centromeric accumulation might be an indirect effect, resulting from loss of such cells from the population, in which Skipper-1 integrated in other genomic positions, as this might cause mutations in the gene-dense genome.

Skipper-1 was previously shown to be under the transcriptional control by DNA methylation, as Skipper-1 transcripts accumulated in the *dnmA* gene deletion strain, resulting in an increase of its genomic copy numbers. Additionally, components of the RNAi machinery appear to control Skipper-1 post-transcriptionally (Kuhlmann et al., 2005). Söderbom and co-workers noticed an extended hairpin derived of a Skipper-1 fragment, which might be the source of the observed small Skipper-1 RNAs (Hinas et al., 2007). Mechanistic details of Skipper-1 integration into centromeric heterochromatin are currently not available, nor models on how the transcriptional and post-transcriptional control mechanisms might be intertwined to result in only two intact Skipper-1 copies in the *D. discoideum* genome (Spaller et al., 2016).

## DIRS-1

Albeit featuring LTRs, the Dictyostelium Intermediate Repeat Sequence (DIRS-1) is the founding member of its own class of TEs as it features a tyrosine recombinase (YR) instead of a canonical integrase (INT) (Cappello et al., 1985; Poulter and Goodwin, 2005). The enzyme is thought to integrate into the genome circular intermediates (Poulter and Goodwin, 2005), the existance of which we recently verified experimentally (Boesler et al., 2014). Full length DIRS-1 contains three, partially overlapping ORFs that are surrounded by two inverted LTRs (Figure 2A). DIRS-1 is the most frequently occurring retrotransposon in *D. discoideum* and this expansion appears unique amongst dictyostelids (Spaller et al., 2016). As seen for Skipper-1, DIRS-1 localizes to centromers (Dubin et al., 2010), of which it constitutes 50% of sequence content (Glöckner and Heidel, 2009). This accumulation has been attributed to the YR, which might facilitate homologous recombination into existing copies (Cappello et al., 1984).

A potentially important feature of DIRS-1 is the internal complementary region (ICR; Figure 2A), a non-coding sequence that displays complementary to the 5′ end of the left LTR and to the 3′ end of the right LTR (Cappello et al., 1985; Poulter and Goodwin, 2005). DIRS-1 is transcriptionally active (Cappello et al., 1985) and like for many other retroelements, its LTR sequences serve as promoters (Wiegand et al., 2014), a feature that was recently applied in a knock-down system (Friedrich et al., 2015). For DIRS-1, the inverted orientation of the LTRs results in both sense and antisense transcripts (Wiegand et al., 2014). The sense transcript represents an incomplete copy of DIRS-1, with a small fragment of the left LTR and most of the right LTR missing (Cappello et al., 1985). A mechanism for DIRS-1 replication was proposed (Cappello et al., 1985; Poulter and Goodwin, 2005), but so far experimentally not fully proven. In this, the missing LTR sequences would be reconstituted by using the complementary ICR as template during cDNA synthesis. Upon self-ligation and formation of circular cDNA, a double-stranded molecule would be generated, allowing for site-specific recombination. The last step of this model is indirectly supported by DIRS-1 preferentially targeting existing genomic copies of itself, without apparent sequence preference (Cappello et al., 1984).

Unlike Skipper-1, DIRS-1 appears to be exclusively under post-transcriptional control by components of the RNAi machinery, in particular the RdRP RrpC and the argonaute AgnA (Boesler et al., 2014; Wiegand et al., 2014). In the absence of these two proteins, the amounts of endogenous small (21mer) DIRS-1 RNAs are largely reduced, concurrent with an accumulation of full length and shorter DIRS-1 mRNAs. Southern blot analysis suggested novel DIRS-1 integrations as consequence of the missing post-transcriptional silencing. The small DIRS-1 RNAs observed in the wildtype represent the majority of the small RNA population in *D. discoideum* (Hinas et al., 2007). They are asymmetrically distributed over the DIRS-1 element, and in particular the region of ORF1 appears devoid of significant amounts of small RNAs (Wiegand et al., 2014). As a consequence, GFP fusions of only ORF1, but not of the other two ORFs are translated in the wildtype, while all three GFP fusions can be readily obtained in strains lacking RrpC or AgnA (Boesler et al., 2014; Wiegand et al., 2014). The molecular phenotype with respect to DIRS-1 thus appears to be highly similar in strains lacking these two proteins. The presence of a circular cDNA copy, that is part of the proposed replication mechanism (Cappello et al., 1985; Poulter and Goodwin, 2005), however, has so far only been experimentally shown for an *agnA* gene deletion strain (Boesler et al., 2014), but not yet addressed in strains lacking RrpC.

## Future perspectives

Based on the highly successful TRE5-A^bsr^ element (Siol et al., 2011), we suggest that similar constructs might be instructive in studying Skipper-1 and DIRS-1 (Figure 2B). To clone the consensus sequence of either element might represent a challenge due to the A/T-richness of the *D. discoideum* genome. In line with this, Siol et al. observed instability of tagged TRE5-A.1 sequences on plasmids (Siol et al., 2011). Potentially, however, this might be overcome by gene synthesis. While we had reported DIRS-1 and Skipper-1 transcript accumulation in respective mutant strains (Kuhlmann et al., 2005; Boesler et al., 2014; Wiegand et al., 2014), this is not necessarily indicative for retrotransposition competence.

Having tagged versions (Figure 2B) would not only allow to investigate whether the elements can perform full transposition cycles in wildtype and RNAi mutant strains. Additionally, the functional relevance of specific sequence elements in the individual retrotransposons could be addressed. For DIRS-1, such experiments might employ a tagged element lacking the ICR (Figure 2A), to address its requirement for the generation of a full length circular cDNA (Cappello et al., 1985; Poulter and Goodwin, 2005; Boesler et al., 2014). Likewise, the functionality of the two Skipper CHDs might be investigated (Figure 2A) to determine if they are important for the observed integration sites.

Finally, also novel DIRS-1 and Skipper-1 integration sites might be mapped (Figure 2C). For this, the sequence of the inserted resistance cassette might be experimentally addressed by Southern blotting or inverse PCR (Figure 2C), thereby discriminating between old and novel integration sites.

## Conclusion

The contemporary retroelements present in the genome of *D. discoideum* are all found in comparably safe integration sites, either in the vicinity of tRNA genes, or in centromeric sequences, thereby largely preventing mutational insertions. Presumably, the gene-dense genome of the amoeba did not tolerate any retrotransposon with lacking integration specificity during evolution. The three best-studied retroelements, TRE5-A, Skipper-1, and DIRS-1, representing the major retroelement classes of this amoeba (Figure 2A), are all cellularly controlled by distinct components of the RNAi machinery. This points towards tailor-made cellular responses to the idiosyncrasies of the individual retrotransposon. The observation that the RNAi component AgnC, which acts in the regulation of TRE5-A, is itself regulated by a host factor that is involved in TRE5-A retrotransposition, points toward a complex, interacting control network, rather than a linear control featuring two components. Whether similar control networks exist also for other retrotransposons stands to be determined in future work.

## Author contributions

MM and MI drafted the manuscript and designed the figures. CH wrote the manuscript.

### Conflict of interest statement

The authors declare that the research was conducted in the absence of any commercial or financial relationships that could be construed as a potential conflict of interest.
